# Research on the local path planning of an orchard mower based on safe corridor and quadratic programming

**DOI:** 10.3389/fpls.2024.1403385

**Published:** 2024-11-01

**Authors:** Jun Li, Haomin Li, Ye Zeng, Runpeng Jiang, Chaodong Mai, Zhe Ma, Jiamin Cai, Boyi Xiao

**Affiliations:** ^1^ College of Engineering, South China Agricultural University, Guangzhou, China; ^2^ Guangdong Laboratory for Lingnan Modern Agriculture, Guangzhou, China; ^3^ State Key Laboratory of Agricultural Equipment Technology, Beijing, China

**Keywords:** mower robot, safe corridor, path planning, quadratic programming, jerk

## Abstract

**Introduction:**

Path planning algorithms are challenging to implement with mobile robots in orchards due to kinematic constraints and unstructured environments with narrow and irregularly distributed obstacles.

**Methods:**

To address these challenges and ensure operational safety, a local path planning method for orchard mowers is proposed in this study. This method accounts for the structural characteristics of the mowing operation route and utilizes a path-velocity decoupling method for local planning based on following the global reference operation route, which includes two innovations. First, a depth-first search method is used to quickly construct safe corridors and determine the detour direction, providing a convex space for the optimization algorithm. Second, we introduce piecewise jerk and curvature restriction into quadratic programming to ensure high-order continuity and curvature feasibility of the path, which reduce the difficulty of tracking control. We present a simulation and real-world evaluation of the proposed method.

**Results:**

The results of this approach implemented in an orchard environment show that in the detouring static obstacle scenario, compared with those of the dynamic lattice method and the improved hybrid A* algorithm, the average curvature of the trajectory of the proposed method is reduced by 2.45 and 3.11 *cm* ^–1^, respectively; the square of the jerk is reduced by 124 and 436 *m* ^2^/*s* ^6^, respectively; and the average lateral errors are reduced by 0.55 *cm* and 4.97 *cm*, respectively, which significantly improves the path smoothness and facilitates tracking control. To avoid dynamic obstacles while traversing the operation route, the acceleration is varied in the range of -0.21 to 0.09 *m*/*s* ^2^. In the orchard environment, using a search range of 40 *m* × 5 m and a resolution of 0.1 *m*, the proposed method has an average computation time of 9.6 *ms*. This is a significant improvement over the open space planning algorithm and reduces the average time by 12.4 *ms* compared to that of the dynamic lattice method, which is the same as that of the structured environment planning algorithm.

**Discussion:**

The results show that the proposed method achieves a 129% improvement in algorithmic efficiency when applied to solve the path planning problem of mower operations in an orchard environment and confirm the clear advantages of the proposed method.

## 1 Introduction

Weed control is a crucial aspect of orchard production. Grass infestation can have adverse effects on soil nutrients, the growing space of orchard crops, and light availability, ultimately leading to a reduction in fruit tree yield (Hu et al., 2023). Using mowers with an autonomous navigation system can significantly enhance the efficiency of mowing operations compared to the traditional manual method (Bai et al., 2023; Rai et al., 2023; Thakur et al., 2023). Path planning is the foundation for achieving autonomous navigation of lawn mowers. The objective of this strategy is to compute an optimal collision-free operation path that meets constraints while minimizing operation costs, such as total operation distance, operation time, and energy consumption. Reasonable path planning algorithms ensure operational safety, reduce the total operating path length and excess coverage, and improve the operational efficiency of mowers. Promoting the standardization and normalization of agricultural production methods is significant for efficient smart agriculture (Yang et al., 2023; Fasiolo et al., 2023).

During normal operations, the mower follows a predetermined global route. However, when a collision risk is detected, local path planning is performed to ensure a collision-free and feasible time sequence that meets kinematic constraints and avoids obstacles. This is achieved without deviating excessively from the global route or exceeding the boundaries of the operating area (Chengliang et al., 2020). Local path planning algorithms can be classified into several categories, including graph search-based, sampling-based, curve interpolation fitting-based obstacle avoidance, artificial potential field-based, reinforcement learning-based, and numerical optimization-based local path planning (Bloch et al., 2018; Ren et al., 2020; Zhong et al., 2020; Hu et al., 2021; Wang et al., 2021; Zhang et al., 2022; Zhuang et al., 2023). Graph search-based methods, such as the A* algorithm and the state lattice algorithm, are capable of handling high-dimensional data and are suitable for local planning in dynamic environments. However, these methods are computationally expensive and have limitations in discrete resolution. On the other hand, sampling-based methods perform well in high-dimensional spaces but are prone to expansion failure in narrow environments and tend to generate overly aggressive planning trajectories (Yang and Lin, 2021). Curve-based methods can generate smooth trajectories, but they are usually computationally expensive (Xi et al., 2019; Cheng et al., 2022; Yang et al., 2022). Optimization-based methods, such as those used by Dmitri Dolgov et al. (Dolgov et al., 2010), can improve the quality of existing paths. In their study, hybrid A* trajectories were optimized using numerical nonlinear functions, which performed well in unstructured and complex environments. The solution time was controlled in the range of 50-300 ms. The search problem can be modeled as an optimization problem, in which various constraints, such as the velocity, acceleration, and minimum steering radius, are integrated into a unified model for problem solving. This approach is widely used in autonomous driving and robotics systems due to its ability to handle dynamic obstacles and different types of constraints. The proposed method also utilizes an optimization-based approach.

**Figure 1:**
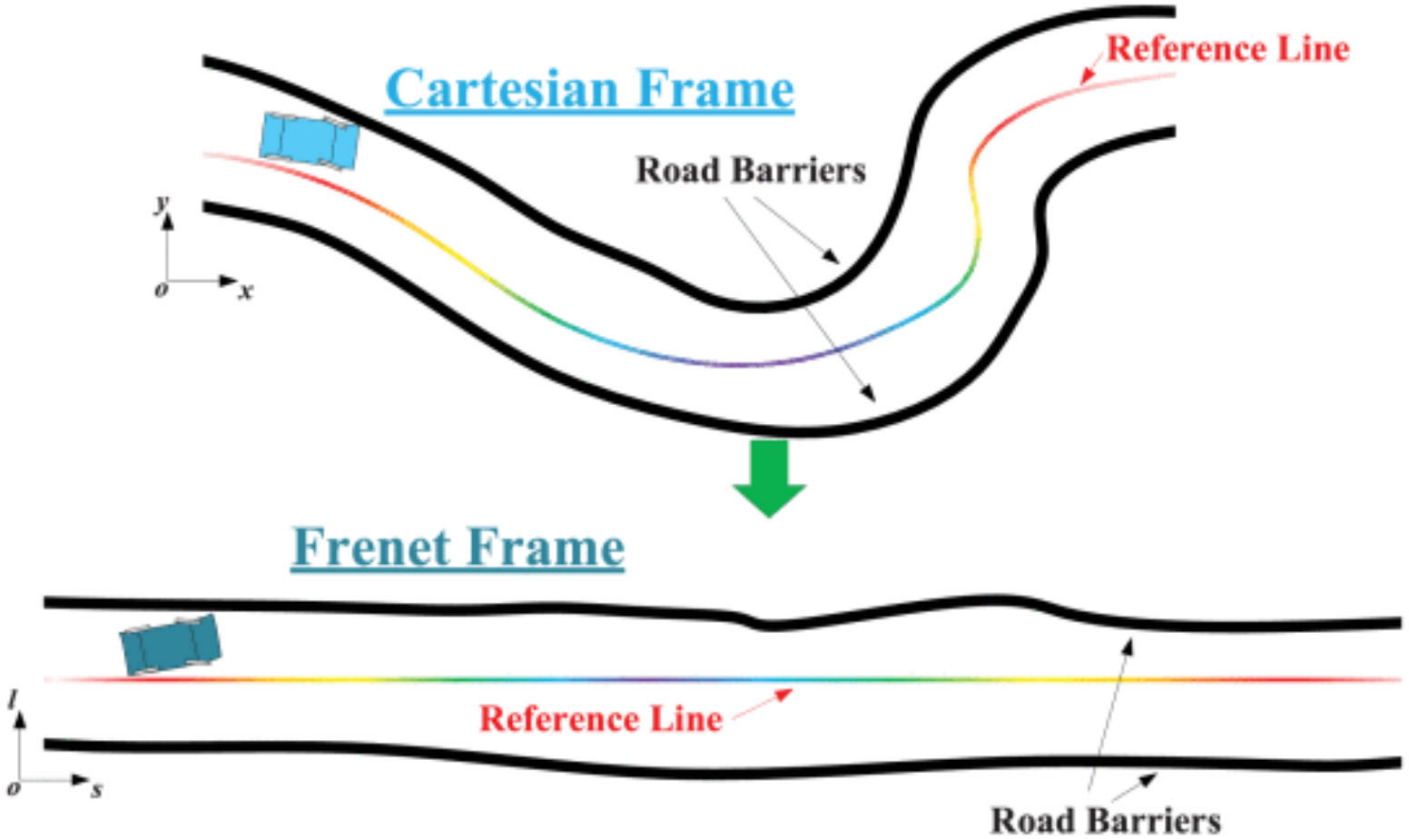
Schematic diagram of the conversion from Cartesian to Frenet frames.

In a standardized orchard, fruit trees are distributed according to specific rows and plant spacings, resulting in a highly structured operation path. Weeds typically grow between the rows of fruit trees, which creates a parallel distribution of the operation area boundary and the operation path, also known as the global reference route. Therefore, for mowing operations, algorithms that are applicable to structured environments are more advantageous than open-space algorithms. Optimization-based methods are commonly used in structured scenarios with existing reference lines. In addition to path planning methods in the Cartesian frame, some approaches transform the planning problem to different dimensions to reduce complexity. The Frenet frame is commonly utilized for trajectory planning in structured environments (Werling et al., 2010). As depicted in **Figure 1**, irregularly shaped reference lines in the Cartesian frame are transformed into straight reference lines in the Frenet frame. This approach has the advantage of normalizing any road to a straight tunnel with left and right boundaries. Consequently, the nonlinear obstacle avoidance constraints in the trajectory planning problem are converted into linear in-channel constraints. Furthermore, the motion constraints that were originally coupled are now decoupled into independent forms in both the longitudinal and lateral directions (Li et al., 2016). This reduces the planning dimension. The Frenet frame-based method allows for the description of the trajectory planning problem as an optimization problem, specifically quadratic programming (QP), which can be solved quickly (Wang et al., 2020; Lim et al., 2021). Optimization-based methods that utilize the Frenet frame are commonly employed in industry (Paden et al., 2016; Zhang et al., 2022). Lim et al. (2018) employed a hierarchical trajectory planning algorithm that integrated a sampling-based behavioral planner and an optimization-based motion trajectory planner to facilitate autonomous driving in urban environments. Fan et al. (2018) utilized a combination of dynamic programming and quadratic programming to generate path and speed profiles by lateral and longitudinal decoupling in the Frenet frame. They obtained a rough solution using dynamic programming to create a convex space for the solution and then used quadratic programming to solve the problem with second-order convergence of the speeds. This method was applied to Baidu’s self-driving car and was successfully tested on highways and urban roads in Beijing. However, agricultural scenes are mostly unstructured, resulting in fewer applications in agriculture. Yang Lili et al. (Fan et al., 2018) proposed a method for behavioral decision-making and real-time local path planning for agricultural machines in the Frenet frame. The method is designed for real-time obstacle avoidance and speed planning in machinist trials. Trajectory behavioral decision-making and speed behavioral decision-making methods were developed using a finite state machine and an improved dynamic lattice method. The algorithms’ redundancy was reduced, and their timeliness was improved. However, the dynamic lattice method has limitations due to its searching resolution, which allows for only suboptimal solutions. Additionally, multiple collision detections are required at each layer, which limits the computational efficiency of the algorithm.

One of the main challenges of optimization-based methods is obtaining the optimized constraint space, which includes kinematic constraints and obstacle avoidance. In the proposed method, a safe corridor is utilized to represent the obstacle constraint space. In several approaches, safe corridors (SCs) are utilized to plan and avoid static obstacles for autonomous mobile robots, including humanoid robots (Yang et al., 2024), quadrotors (Banerjee et al., 2015), and ground robots (Liu et al., 2017). A safety corridor consists of a series of overlapping convex shapes that can be effectively used by existing optimization solvers for robot planning due to their convex enveloping nature. These safety corridors are generated around a reference path that does not consider the system’s dynamics. The generation method typically involves sampling a given path and creating convex shapes around the sampling points or path segments. Within the safety corridor, dynamically feasible trajectories are generated to avoid collisions with static obstacles. A planning method using safe corridors (Liu et al., 2017; Tordesillas et al., 2022) has recently been proposed and compared with other state-of-the-art methods. It has been proven to be superior in terms of computation time, trajectory speed, and trajectory smoothness. The trajectories are confined to safe corridors, ensuring collision-free movement. Safe corridors are well suited for integration with optimization-based methods. The key is to improve the quality of the safe corridor generator in terms of computation time.

Existing studies on obstacle avoidance in agricultural scenarios are often based on open space planning algorithms in unstructured environments. However, in these studies, deviations from reference routes and operational boundary constraints are often not considered. Additionally, the increase in search dimensions and search space can reduce the efficiency and optimality of algorithmic solutions. It is important to address these issues in future research. Furthermore, the high-order derivative terms of the path, such as the curvature and jerk, which significantly affect the driving stability, are often overlooked. Although structured algorithms are used in agricultural scenarios, they still have deficiencies in terms of computational efficiency and path quality.

To address the aforementioned challenges and account for the structured nature of mowing operation routes, a local path planning algorithm that utilizes safe corridors and quadratic programming is presented in this paper. The algorithm introduces two key innovations.

1. A depth-first search method is employed to rapidly construct a safe corridor, which can then be used to determine the detour direction and provide a convex space for the optimization algorithm.
2. Piecewise constraints on jerk and curvature are introduced into the quadratic planning process. This ensures higher-order continuity and curvature feasibility of the paths and reduces the difficulty of tracking control.

The method described has been tested in simulations with narrow scenarios and has been implemented on hardware for real-vehicle testing in an orchard. The results of both simulation and real vehicle tests demonstrate that the proposed method significantly improves computational efficiency when compared to the open space planning algorithm (Dolgov et al., 2010) and the dynamic lattice method (Zhou et al., 2022), which is also a structured environment planning algorithm. The results validate the advantages of the proposed method in addressing the path planning problem of mowing operations in an orchard environment.

The rest of this article is organized as follows. We give an overview of the complete system pipeline and the details of each key components in Sections 2. The benchmark comparison on simulation map and the real-world experiments are reported in Section 3. Finally, Section 4 concludes this article.

## 2 Local path planning method based on safe corridors and quadratic programming

**Figure 2:**
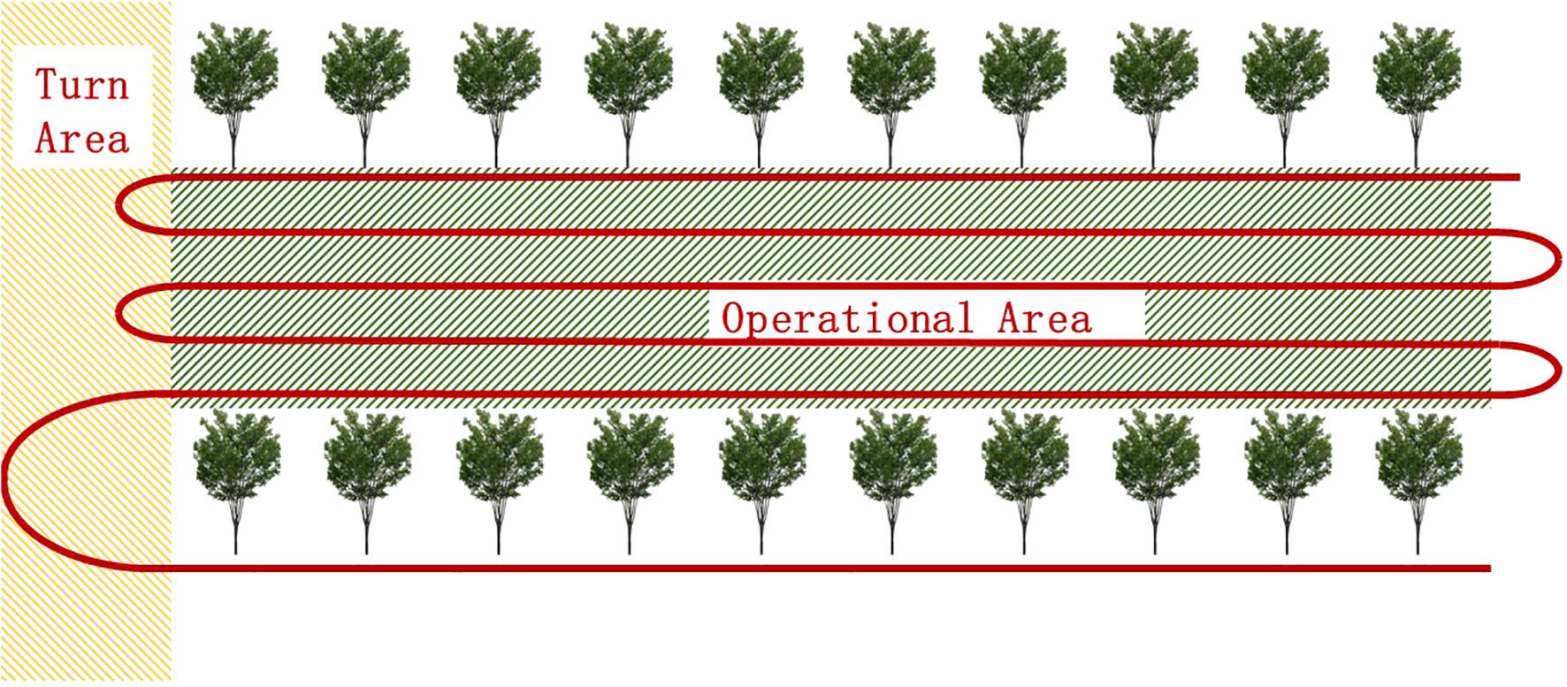
Schematic diagram of the operation route.

In this paper, the current motion state (x,y,θ,κ,v,a) of the lawn mower is laterally and longitudinally divided with the help of a reference line in trajectory planning. The global reference line and the operation boundary are typically parallel distributed straight lines instead of curves in the usage scenario of this paper, as shown in **Figure 2**. No iterative optimization is needed. Thus, the Cartesian frame can be used to represent the lateral and longitudinal motions directly, reducing the planning dimension with the aid of reference lines in the XY grid instead of the Frenet frame.

The operational area is situated amidst the rows of fruit trees, and the red path serves as the global reference route for operations.

In this paper, a path-speed decoupling approach is proposed to separate path planning and speed planning. Moreover, static obstacles are considered in path planning, and a speed profile is generated to avoid dynamic obstacles based on the initial trajectory. While the path-speed decoupling method may not be optimal for dynamic obstacles, it offers great flexibility in both path and speed planning and has higher computational efficiency.

Path planning is initially based on the depth-first search method. The decision on the detour direction is made by constructing a safe corridor. Once the convex solution space is obtained, piecewise optimization is performed via quadratic programming to derive a smooth original trajectory that can avoid static obstacles. In speed planning, when planning for low-speed dynamic obstacles, we refer to the method described in the literature (Paden et al., 2016). We make the decision on obstacle avoidance behavior by finding an optimal piecewise speed profile on the XT grid using dynamic programming. We then optimize it using quadratic programming, resulting in a feasible and smooth speed profile. For high-speed dynamic obstacles, we prioritize immediate stopping for safety reasons rather than obstacle avoidance.

### 2.1 Method for generating safety corridors using a depth-first search algorithm

Our approach determines the objectives and constraints for path optimization based on the generated reference line. In this section, the method for generating feasible search regions for the optimization process is explained. To ensure convergence to an optimal solution, the cost function must be convex, requiring the optimization matrix of the model to be semi-positive definite, i.e., the space to be solved must be convex. The feasible region of the path may consist of multiple geometric spaces separated by obstacles. For example, passing a static obstacle from the left or right will create a two-way direction around the obstacle. At this point, the optimal path and speed solutions are still in the nonconvex space, and a method is needed to make decisions about the bypass direction. In this paper, we propose a decision-making strategy that utilizes depth-first search to search for safe corridors in an entire operation area. The strategy accounts for the operation boundary, the positions of static obstacles and the geometric information. Therefore, inequality constraints are determined for use in later optimization steps. This approach enables the creation of convex feasible spaces. As a result, a quadratic programming-based smooth spline curve solver can produce smoother path and speed profiles that adhere to this decision.

Algorithm 1 **Safe Corridor Search Algorithm.**

**Figure 3:**
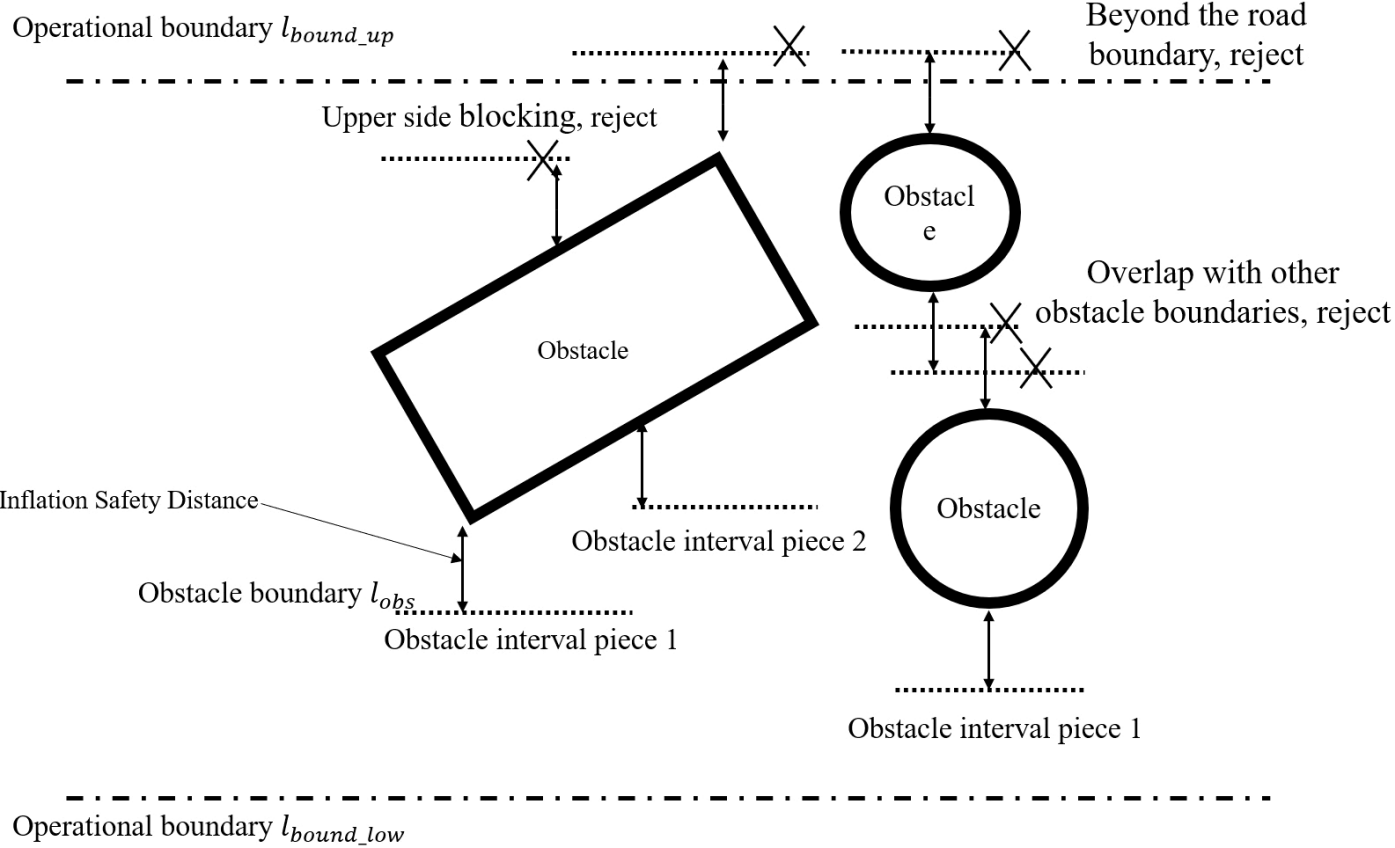
Schematic diagram of boundary generations.

** Parameters**: Preset resolution Δ*s*
1 *s* ← discretize the vertical displacement space along the reference line to the preset resolution Δ*s;*
2 *l_bound_
* ← generate job road boundary;
3 *l_obs_
* ← generate obstacle boundary;
4 **for** *each s_i_
* **in** *s* **do**
5 **if** *exists obstacle boundary(s_i_)* **then**
6  **if** *is detour longitudinal interval(s_i_)* **then**
7   *detour_directions* ← sort(all detour directions(*s_i_
*));
8   **for** *each direction* **in** *detour_directions* **do**
9    **if** *search fails(s_i_, direction)* **then**
10     **continue**;
11    **endif**
12    **else**
13     **break**;
14    **endif**
15   **endfor**
16  **endif**
17  **else if** *is single row longitudinal interval(s_i_)* **then**
18   **continue**;
19  **endif**
20 **endif**
21 **else**
22  *l_low_
*, *l_up_
* ← *l_bound_
* (*s_i_
*);
23 **endif**
24 **endfor**
25 **return** *l_bound_
*;

**Algorithm 1** shows the process for identifying safe corridor boundaries for mower accessibility. The space for longitudinal displacement is discretized to a predetermined resolution along the reference route. Then, operational road boundaries and obstacle boundaries are generated. The operational road boundary, $l_{bound}\left(s_{i}\right)$ , is the lateral boundary of the current operational area at each point. It usually coincides with the rows of fruit trees. Only static obstacles within the rectangle formed by the search distance and the operational road boundary are considered in the obstacle boundary. It expands outward by an appropriate safety distance. Obstacles are typically irregular edges, and regularization is carried out at an appropriate resolution, $\text{Δ}s_{ob}$ , to obtain one or more pairs of lateral obstacle boundaries, $l_{obs}\left(s_{i}\right)$ , and each pair of lateral obstacle boundaries contains upper and lower boundaries located on the upper and lower sides of the obstacle, respectively, as shown in **Figure 3**. If an obstacle boundary extends beyond the operational roadway boundary or overlaps with other obstacle boundaries, it is considered a non-detourable direction, and all boundaries of the obstacle on that side are removed. The intervals along the longitudinal axis where obstacles are present are referred to as obstacle intervals. When there are two or more obstacle boundaries within a longitudinal interval, it is called a detour longitudinal interval. On the other hand, when there is only one obstacle boundary on a side, it is referred to as a one-way longitudinal interval, and there is no need to decide on a detour direction. If there are obstacles present but no obstacle boundaries, a complete blockage occurs, resulting in a failed search.

The length of an obstacle interval segment cannot exceed $\text{Δ}s_{ob}$ . If the obstacle occupies less than $\text{Δ}s_{ob}$ units longitudinally, the segment length is equal to the obstacle’s longitudinal length. If the obstacle’s longitudinal occupation exceeds $\text{Δ}s_{ob}$ , multiple obstacle interval segments exist.

**Figure 4:**
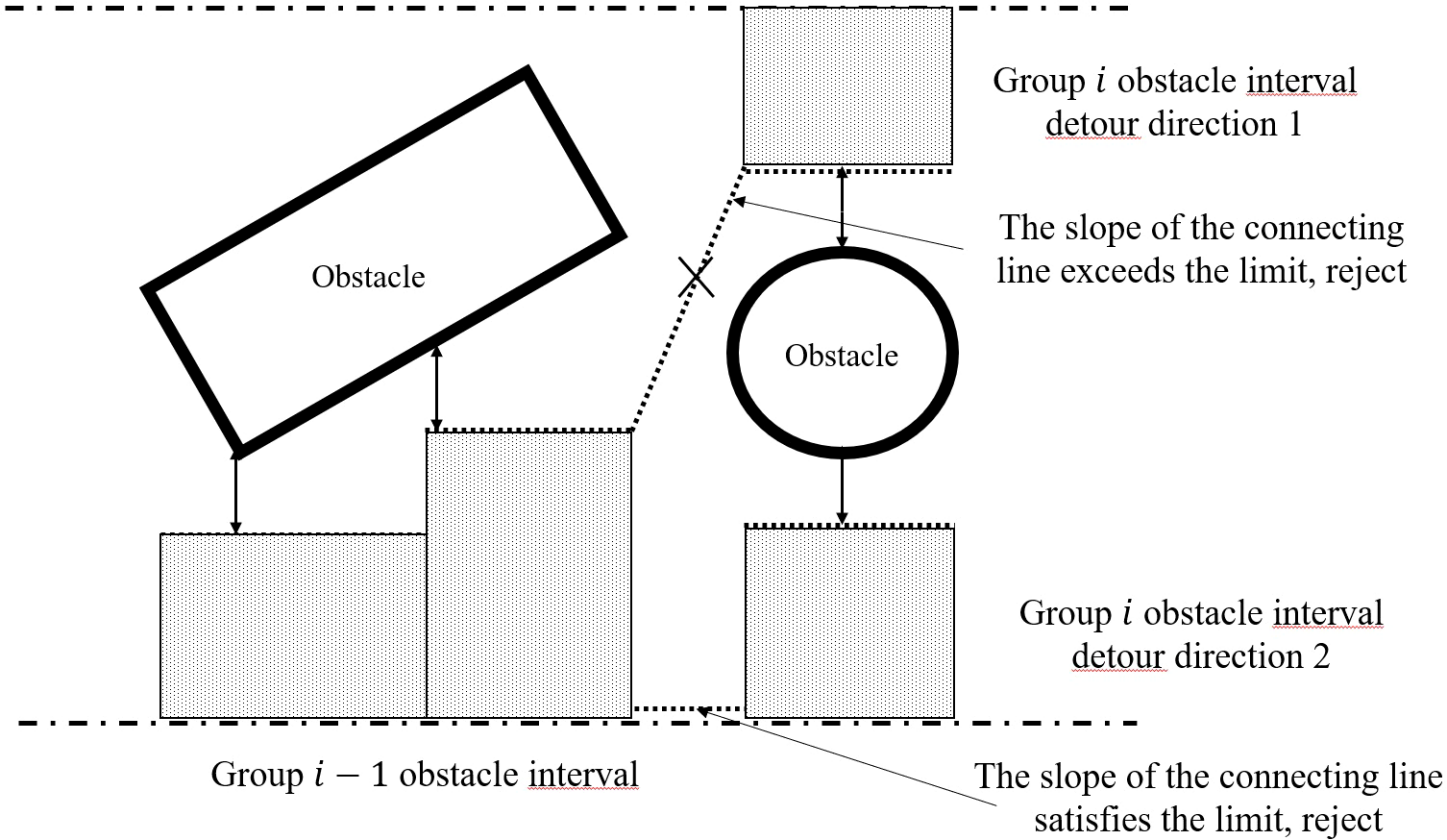
Schematic diagram of the detour direction decisions.

To determine the safe corridor boundary, we first search forward from the starting point, $s_{0}$ . If there is no obstacle boundary at $s_{i}$ , we use the operational road boundary as the lower boundary of the safe corridor at $s_{i}$ . We then continue to the next point. However, if there is an obstacle boundary at $s_{i}$ , we employ a depth-first search method:

First, a feasibility assessment is carried out based on the lateral acceleration limit of the chassis. There is a limit to the rate of change of lateral displacement with constant longitudinal speed. Therefore, the most backward boundary of the interval is connected to the most forward boundary of the previous obstacle interval, and the slope of the line is evaluated. If the slope of this line is outside the acceptable range or the connection line cannot avoid the obstacle, the current interval is deemed unreachable, as shown in **Figure 4**. If there are additional detour options, the next direction is searched. Otherwise, the search is unsuccessful. After the feasibility assessment, if it is a one-way longitudinal interval, we skip to the next $s_{i}$ . If it is a detour longitudinal interval, we sort all the detour directions by their lateral widths. We then start with the widest detour direction and search for subsequent $s_{i}$ . If the search along this direction fails, we gradually backtrack and try other directions. This process is repeated until complete blockage or until the maximum search range of 15 meters or 15 seconds of travel in our implementation is reached.

Concatenation is conducted to select the closest boundary of the two obstacle intervals, which can be the upper boundary, the lower boundary, or the center. Using this figure as an example, Detour Direction 1 is closest to the previous interval as the lower boundary, and the previous interval is closest to Detour Direction 1 as the upper boundary. Similarly, Detour Direction 2 is closest to the previous interval as the lower boundary, and the previous interval is closest to Detour Direction 1 as the lower boundary.

**Figure 5:**
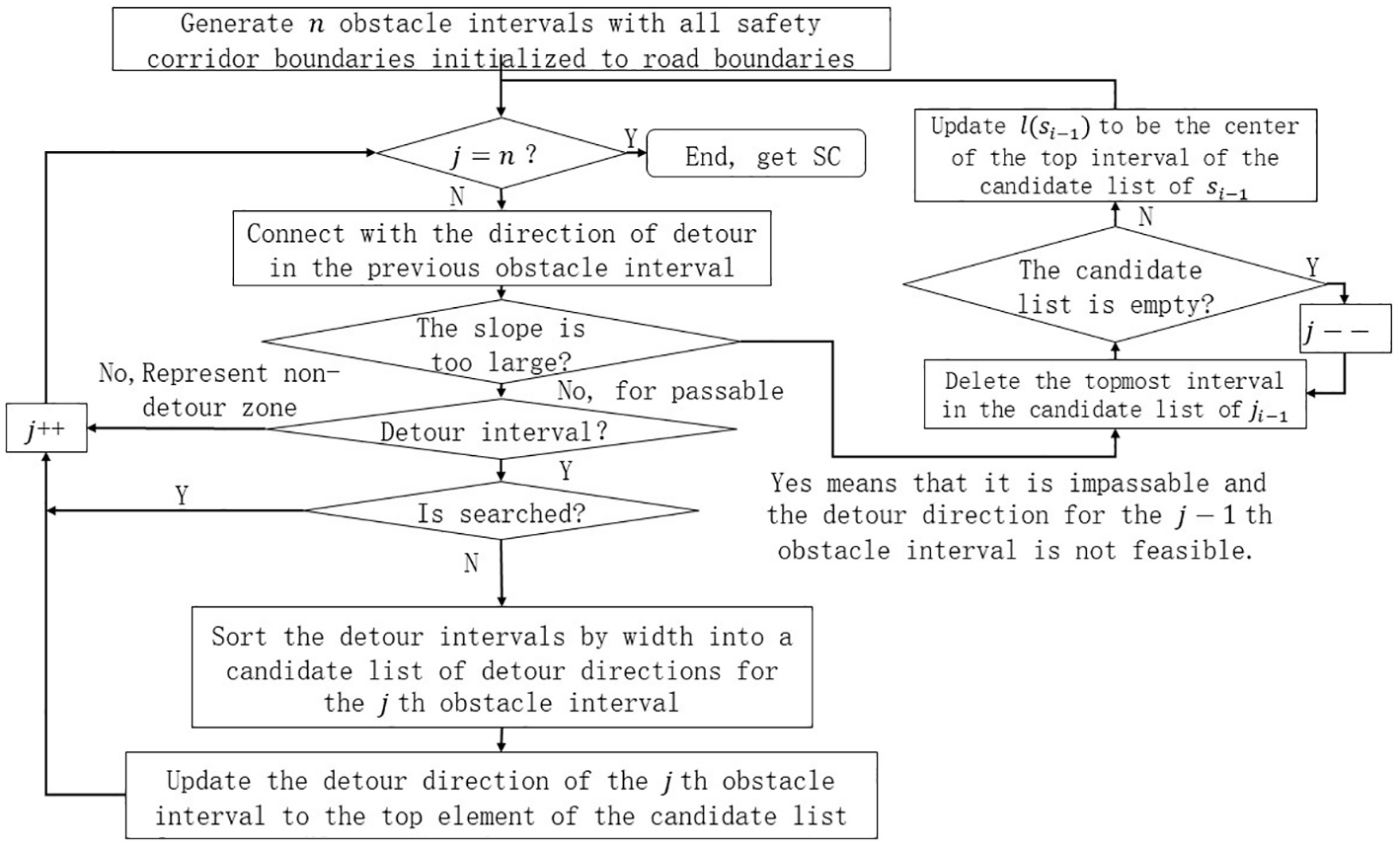
Flow chart for safe corridor generation.

The output of the depth-first search described above is a feasible region for all sampling points. Backtracking from the endpoints yields a series of safe corridors consisting of operational road boundaries, one-way intervals, and detour intervals. The method produces a function that maps the safe corridor boundaries, $\left(y_{low},y_{up}\right)$ , to each longitudinal location, s . The overall flow of the algorithm is shown in **Figure 5**.

### 2.2 Path optimization method based on piecewise jerk

In this section, we establish the optimization model and use the piecewise step-by-step construction method to optimize the obstacle avoidance trajectory using quadratic programming based on current state. The safe corridor boundaries calculated in Section 2.1 are also considered.

The path optimization model has the following several constraints that must be satisfied:

1. The lawnmower must remain within the boundaries of the operating area and avoid any collisions with obstacles.
2. There are specific kinematic constraints on the lateral speed, lateral acceleration, and lateral plus acceleration of the mower depending on the current state.
3. All kinematic relationships must be satisfied.

The optimization metrics considered are as follows:

1. There must be no collisions. It is crucial that the path does not intersect with any obstacles in the environment, and a buffer space must be reserved to ensure this condition.
2. When there is no risk of collision, the mower should follow the reference operating route as closely as possible.
3. The lateral deviation should be minimized as much as possible. The rate of change of lateral displacements, as well as their accelerations and jerks, should be minimized. To facilitate trajectory tracking control, it is recommended to use lower lateral speeds and their higher-order derivatives.
4. The distance between the mower and the obstacle should be maximized. To ensure the safe passage of the mower, it is important to maximize the distance between the mower and any obstacles. The metric for this is the distance between the mower and the corresponding safety corridor boundary. This can be achieved by using safety corridors, which provide a safe and feasible space.

To summarize, the cost function for optimizing path, y(x) , construction is determined by the weighting of the metrics mentioned above.


$$f\left(y\left(x\right)\right)=w_{y}*\int y{\left(x\right)}^{2}dx+w_{y^{\prime }}*\int y^{\prime }{\left(x\right)}^{2}dx$$

(1) $$+w_{y^{\prime \prime }}*\int y^{\prime \prime }{\left(x\right)}^{2}dx+w_{y^{\prime \prime \prime }}*\int y^{\prime \prime \prime }{\left(x\right)}^{2}dx$$

$$+w_{obs}*\int {\left(y\left(x\right)-0.5*\left(y_{bound}{\left(x\right)}_{min}+y_{bound}{\left(x\right)}_{max}\right)\right)}^{2}dx$$

The weight coefficients for lateral deviation, lateral movement speed, acceleration, and jerk cost are represented by $w_{y}$ , $w_{y^{\prime }}$ , $w_{y^{\prime \prime }}$ , and $w_{y^{\prime \prime \prime }}$ , respectively. The objective is to minimize these factors. Additionally, $w_{obs}$ represents the weight coefficient for the obstacle distance cost, and the objective is to maximize the distance from the obstacle. In the above cost function, the offset from the safety corridor boundary is relatively more important. A cost function has been added for the offset indicator, but it is only a soft constraint and cannot limit the specific value of the offset. Therefore, a hard constraint on the amount of safety corridor offset has been added:


(2) $$\begin{array}{c} y_{bound}{\left(x\right)}_{min}<y\left(x\right)<y_{bound}{\left(x\right)}_{max},\forall x\in \left[0,x_{max}\right] \end{array}$$

The longitudinal coordinates of the safety corridor are indicated by $y_{bound}{\left(x\right)}_{min}$ and $y_{bound}{\left(x\right)}_{max}$ , representing the lower and upper boundaries, respectively.

To formulate the optimization problem and evaluate constraint satisfaction efficiently, a series of densely discretized points along the longitudinal direction of a reference line is used in the piecewise optimization approach. These points represent the path and are used to control its shape, allowing for the assessment of constraint satisfaction. The main concept is to discretize the one-dimensional lateral displacement function up to the second-order derivable level. Then, a constant third-order derivative term is used to connect two consecutive discrete points to achieve local second-order derivable path smoothness. This approach maintains flexibility and robustness in complex scenarios. The third-order derivative of a position variable is commonly referred to as the jerk, hence the name ‘piecewise jerk method’.


(3) $$\begin{array}{c} \begin{array}{c} y_{0}\\ y_{0}^{'}\\ y_{0}^{\prime \prime } \end{array}\overset{\text{Δ}x}{\to }\begin{array}{c} y_{1}\\ y_{1}^{'}\\ y_{1}^{\prime \prime } \end{array}\overset{\text{Δ}x}{\to }\begin{array}{c} y_{2}\\ y_{2}^{'}\\ y_{2}^{\prime \prime } \end{array}\ldots \begin{array}{c} y_{n-2}\\ y_{n-2}^{'}\\ y_{n-2}^{\prime \prime } \end{array}\overset{\text{Δ}x}{\to }\begin{array}{c} y_{n-1}\\ y_{n-1}^{'}\\ y_{n-1}^{\prime \prime } \end{array} \end{array}$$

The equation above illustrates the discretization of the path function. The variables $y_{i}^{'}$ and ${y_{i}}^{\prime \prime }$ represent the first- and second-order derivatives of $y_{i}$ with respect to the longitudinal coordinates x . Each discrete point of $y_{i}$ , $y_{i}^{'}$ , and ${y_{i}}^{\prime \prime }$ controls the shape of the path and is to be optimized. The piecewise jerk method assumes that consecutive points are connected by a constant third-order term $y^{\prime \prime \prime }$ . The third-order value is calculated by subtracting the second-order value using the finite difference method:


(4) $$y_{i\to i+1}\prime \prime \prime =\frac{y_{i+1}^{\;\prime \prime }-y_{i}^{\;\prime \prime }}{\text{Δ}x}$$

where the third-order term $y_{i}^{\prime \prime \prime }$ is a constant only between two consecutive points, while $y_{i}^{\prime \prime \prime }$ may vary between different consecutive points. To maintain path continuity, an additional equational constraint is introduced between the i th and i+1 th points.


$$y_{i+1}^{\;\prime \prime }=y_{i}^{\;\prime \prime }+\int_{0}^{\text{Δ}x}y\prime \prime \prime dx=y_{i}^{\;\prime \prime }+y_{i\to i+1}\prime \prime \prime *\text{Δ}x$$

(5) $$\begin{array}{c} y_{i+1}^{\;\prime }=y_{i}^{\;\prime }+\int_{0}^{\text{Δ}x}y{\left(x\right)}^{\prime \prime }dx=y_{i}^{\;\prime }+y_{i}^{\;\prime \prime }*\text{Δ}x+\frac{1}{2}*y_{i\to i+1}\prime \prime \prime *\text{Δ}x^{2} \end{array}$$

$$\begin{array}{c} y_{i+1}=y_{i}+\int_{0}^{\text{Δ}x}y{\left(x\right)}^{'}dx\\ \;\;=y_{i}+y_{i}^{\;\prime }*\text{Δ}x+\frac{1}{2}*y_{i}^{\;\prime \prime }*\text{Δ}x^{2}+\frac{1}{6}*y_{i\to i+1}\prime \prime \prime *\text{Δ}x^{3} \end{array}$$

The procedure for solving the piecewise jerk optimization method is as follows: $y_{i},\;y_{i}^{'},\text{ and }{y_{i}}^{\prime \prime }$ must be found within i∈[0,n−1] to minimize the following cost function:


$$f\left(y\left(x\right)\right)=w_{y}*\sum_{i=0}^{n-1}y_{i}^{2}+w_{y^{\prime }}*\sum_{i=0}^{n-1}y_{i}^{\;\prime 2}$$

(6) $$+w_{y^{\prime \prime }}*\sum_{i=0}^{n-1}y_{i}^{\;\prime \prime 2}++w_{y\prime \prime \prime }*\sum_{i=0}^{n-2}{\left(\frac{y_{i+1}^{\;\prime \prime }-y_{i}^{\;\prime \prime }}{\text{Δ}x}\right)}^{2}$$

$$+w_{obs}*\sum_{i=0}^{n-1}{\left(y_{i}-0.5*\left({y^{i}}_{bound\_min}+{y^{i}}_{bound\_max}\right)\right)}^{2}$$

where ${y^{i}}_{bound\_min}$ and ${y^{i}}_{bound\_max}$ denote the lower and upper boundaries of the path boundary corresponding to the i -th longitudinal coordinate after discretization, respectively. The constraints of the optimization problem include the path continuity constraints and the constraints on the path boundary offsets.

The optimization of the path must adhere to the kinematic feasibility constraints of the chassis, in addition to satisfying the geometric continuity and path boundary constraints. The curvature of the path is the most crucial factor for kinematic feasibility. The equation defining the curvature of the path points is determined by the coordinate transformation formula.


(7) $$\begin{array}{c} \kappa =\frac{\left|y^{\prime \prime }\right|}{{\left(1+y^{\prime 2}\right)}^{\frac{3}{2}}} \end{array}$$

To simplify the equation, we assume that the mower is nearly parallel to the reference operating route. This means that the heading angle of the mower is assumed to be in the same direction as that of the reference operating route at the matching point. Therefore, $\text{ΔΘ}=y^{\prime }=0$ .

Furthermore, κ can be approximated as follows:


(8) $$\begin{array}{c} \kappa =\left|y^{\prime \prime }\right| \end{array}$$

The maximum curvature that the chassis can withstand can be calculated using the tracked chassis dynamics model, the track center distance B , and the maximum steering angular velocity $\omega_{max}$ corresponding to the maximum track sliding rate at a certain speed v that keeps the body stable.


(9) $$\kappa_{max}=\frac{1}{B}\cdot \frac{\omega_{max}}{v}$$

The motion feasibility is considered in the optimization process by adding the following linear constraints:


(10) $$\begin{array}{c} By^{\prime \prime }-\frac{\omega_{max}}{v}\leq 0 \end{array}$$

A quadratic programming model is constructed to solve the problem based on the optimization objective cost function and constraints described above. The quadratic programming standard model is as follows:


(11) $$\begin{array}{c} \min f\left(x\right)=\frac{1}{2}x^{T}Px^{T}+q^{T}x \end{array}$$

s.t.lb≤Ax≤ub

In path planning, the optimization variable x are the lateral coordinates y and their first-, second- and third-order derivatives.


(12) $$x={\left[\begin{array}{cccccccccccc} y_{0} & \cdots & y_{n-1} & \dot{y_{0}} & \cdots & \dot{y_{n-1}} & \ddot{y_{0}} & \cdots & \ddot{y_{n-1}} & \overset{\ldots }{y_{0}} & \cdots & \overset{\ldots }{y_{n-1}} \end{array}\right]}^{T}$$

where n is the number of discrete points of the generated path, and the dimension of this optimization problem is 3n. The optimization objective matrix P is constructed based on the cost function.


(13) P=[2wy⋯0⋮⋱⋮0⋯2wy   2wy′⋯0⋮⋱⋮0⋯2wy′   2(wy″+wy‴Δx2)0⋯⋯0 2(−2wy‴Δx2)2(wy″+2wy‴Δx2)0⋯002(−2wy‴Δx2)⋱ ⋮⋮0⋱⋱⋮⋮  ⋱00⋯⋯02(wy″+wy‴Δx2)]

The matrix A that constrains the path continuity, path boundary offset, and curvature is as follows:


(14) A=[1⋯0⋮⋱⋮0⋯1   1⋯0⋮⋱⋮0⋯11⋯⋯0⋮⋱⋮⋮⋱⋮0⋯⋯1  −11⋱⋱−11 −11⋱⋱−11−Δx2−Δx2⋱⋱−Δx2−Δx2−11⋱⋱−11−Δx⋱−Δx−(Δx)23−(Δx)26⋱⋱−(Δx)23−(Δx)26111]

The upper and lower bounds of the constraint are expressed as follows:


(15) $$lb=\left[\begin{array}{c} \begin{array}{c} y_{0l}\\ \vdots \\ y{\left(n-1\right)}_{l} \end{array}\\ \begin{array}{c} {y^{\prime }}_{\;0l}\\ \vdots \\ y{\left(n-1\right)}_{\;l}^{\prime } \end{array}\\ \begin{array}{c} {y^{\prime \prime }}_{\;0l}\\ \vdots \\ y{\left(n-1\right)}_{\;l}^{\prime \prime } \end{array}\\ \begin{array}{c} -\frac{{\left(\text{Δ}x\right)}^{3}}{6}y{\prime \prime \prime }_{bound}\\ \vdots \\ \begin{array}{l} -\frac{{\left(\text{Δ}x\right)}^{3}}{6}y{\prime \prime \prime }_{bound}\\ \begin{array}{c} -\frac{{\left(\text{Δ}x\right)}^{2}}{2}y{\prime \prime \prime }_{bound}\\ \vdots \\ -\frac{{\left(\text{Δ}x\right)}^{2}}{2}y{\prime \prime \prime }_{bound} \end{array} \end{array} \end{array} \end{array}\right],\;ub=\left[\begin{array}{c} y_{0u}\\ \vdots \\ y{\left(n-1\right)}_{u}\\ \begin{array}{c} {y^{\prime }}_{\;0l}\\ \vdots \\ y{\left(n-1\right)}_{\;u}^{\prime } \end{array}\\ \begin{array}{c} {y^{\prime \prime }}_{\;0l}\\ \vdots \\ y{\left(n-1\right)}_{\;u}^{\prime \prime } \end{array}\\ -\frac{{\left(\text{Δ}x\right)}^{3}}{6}y{\prime \prime \prime }_{bound}\\ \vdots \\ -\frac{{\left(\text{Δ}x\right)}^{3}}{6}y{\prime \prime \prime }_{bound}\\ -\frac{{\left(\text{Δ}x\right)}^{2}}{2}y{\prime \prime \prime }_{bound}\\ \vdots \\ -\frac{{\left(\text{Δ}x\right)}^{2}}{2}y{\prime \prime \prime }_{bound} \end{array}\right]$$

The constructed quadratic planning model is input to the OSQP solver. The planning trajectory obtained from the above method contains the position and speed information of the trajectory points, as well as the lateral acceleration and jerk information. The trajectory is input into the speed planning module, and the speed planning is performed according to the position occupied by the dynamic obstacle at each time point calculated by the prediction module to avoid the dynamic obstacle. The computed results of speed planning are assigned to the original trajectory, i.e., the final trajectory that can avoid static and dynamic obstacles is obtained. In the real-vehicle experiments, the LQR control algorithm is used to calculate the optimal control amount based on the gap between the current position and the position of the trajectory matching point, which is input to the motor controller for execution.

## 3 Algorithm test and result analysis

### 3.1 Test content and parameter setting

To assess the effectiveness of the trajectory planning algorithm, we tested it in a simulated multi-obstacle environment that resembles farmland paths. The algorithm was implemented on a self-developed lawn mower platform. The proposed algorithm was tested in an orchard, and the results were analyzed. In this paper, we compare the algorithm presented here with the improved Hybrid A* algorithm described in Reference (Cheng et al., 2022) and the improved dynamic lattice method in Reference (Lim et al., 2021). Hybrid A* is a classical open space planning algorithm widely used in practice, while the dynamic lattice method is an excellent lateral and longitudinal decoupled farm machinery path planning algorithm based on reference lines, exhibiting high path quality and rapid solving speed. All three algorithms are implemented in C++14/Ubuntu18.04. The algorithm presented in this paper is written in-house and solved using the quadratic convex optimizer OSQP. The other two algorithms use open-source code implementation and default settings.

#### 3.1.1 Simulation test scenarios and parameter settings

An AMD Ryzen 5 4500 6-Core Processor with 16 GB of RAM was used for the simulation. To represent obstacles, the occupancy grid of the ROS was implemented. For collision detection, a rectangle with an outer contour size of 1300 mm × 890 mm (length × width) was used to represent the lawnmower. The simulation scenario includes obstacles of varying sizes to test local path planning algorithms in different obstacle avoidance scenarios. The obstacles consist of four static objects with dimensions of 3 m × 2 m, 1 m × 2 m, and 1 m × 1 m. The location and orientation of each obstacle are fixed in the test for comparison purposes.

First, the map of the operation area is established, the reference operation route consists of straight-line trajectory points, the whole length of the operation area is 40 m, the width is 5 m, the starting coordinates of the agricultural machine are (0, -0.1), the endpoint coordinates are (40,0), and the single planning cycle is 100 ms, ignoring the control error. The parameters in the test are set as follows: the maximum acceleration is 0.6 m/s^2, the operating speed is set to a constant 1 m/s, the maximum curvature limit is 0.21 $m^{-1}$ , the maximum slope of the interval connecting line is 3.8, and the lateral expansion distance is 0.5 m. The optimization weights are set as follows: $w_{y}$ is 0.01, $w_{y^{\prime }}$ is 0.5, $w_{y^{\prime \prime }}$ is 3, $w_{y^{\prime \prime \prime }}$ is 0.1, and $w_{obs}$ is 0.2.

#### 3.1.2 Orchard test scenarios and parameter settings

A self-developed lawn mower platform based on a TK-52 tracked chassis was utilized to conduct a realistic real-machine verification test of the algorithms in this paper.

**Figure 6:**
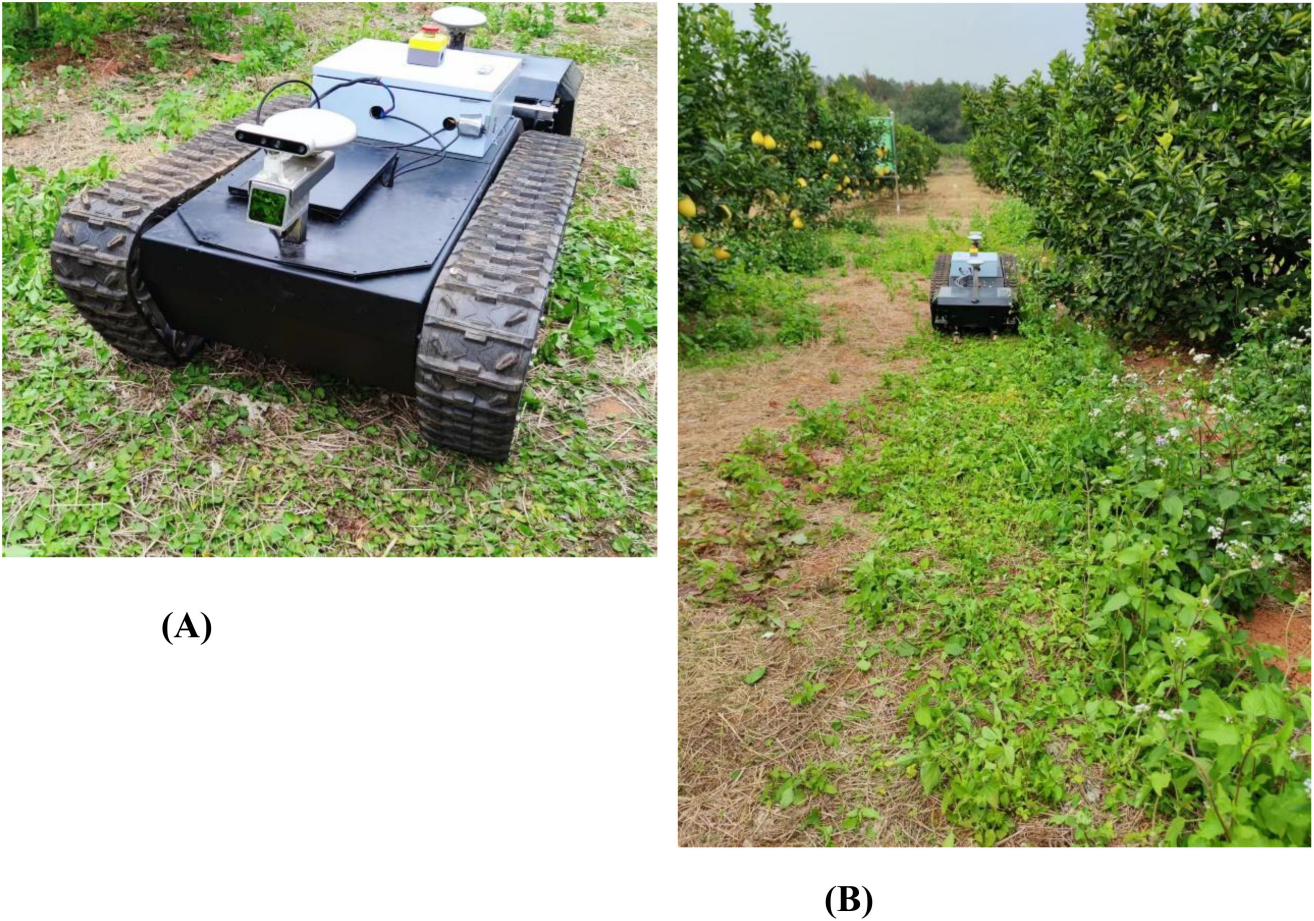
Physical map of the orchard trial. **(A)** Mower platforms. The mower has a traction layout with a tracked undercarriage towing the rear mower module and a white electrical control box at the rear that contains the electrical components of the undercarriage and mower module. **(B)** Test environment.

The semisolid-state LiDAR LIVOX MID 70 can sense the environment around the mower and fuse the IMU high-frequency position information to realize obstacle sensing and localization. High-precision GNSS antennas at the front and rear of the vehicle provide the coordinates of the global reference route. The mower receives the real-time wheel speed through the adaptive encoder and feeds it back to the path tracking module. The LQR control algorithm is used to calculate the optimal control quantity based on the gap between the current position and the position of the trajectory matching point, which is input to the motor controller for execution. The experimental environment is a modern standard orchard in the school, as shown in **Figure 6A**, with a spacing of 4 meters and a length of 25 meters. The real machine platform is shown in **Figure 6B**.

The obstacle and environment settings are the same as those designed in Section 3.1, and pedestrians crossing the operation route with a size of 1 m×1 m and both longitudinal and lateral speeds of 1 m/s are added to serve as dynamic obstacles whose locations and sizes are given by the prediction module, avoided by the speed planning module, and mapped in the XT map.

The algorithms are run on an NVIDIA Jetson NX-based industrial computer with an NVIDIA Carmel ARM^®^v8.2 hardware configuration and 8 GB of RAM.

### 3.2 Test results and analysis

In the experiments, we concentrate on path deviation, average curvature, integral of jerk², and computation time for planning results. Path deviation is defined as the distance between the path and the line y = 0. A smaller distance indicates a lesser deviation from the operational path, thereby focusing more on the operational area. The term “average curvature” is used to describe the mean value of the curvature of the planning result. A reduction in the average curvature results in a smoother transition in steering angular velocity, which in turn makes control and navigation more straightforward and reduces the likelihood of contact with the ground. The integral of jerk² thus represents the degree of aggressiveness of the lateral speed. The computation time is a crucial parameter for assessing the efficacy of an algorithm. A reduction in computation time allows the mower to respond to unforeseen circumstances in a prompt manner. Furthermore, in the real machine test, we also prioritize the assessment of control error, as the quality of the planning results can significantly influence the control effect.

#### 3.2.1 Simulation test results and analysis

**Figure 7:**
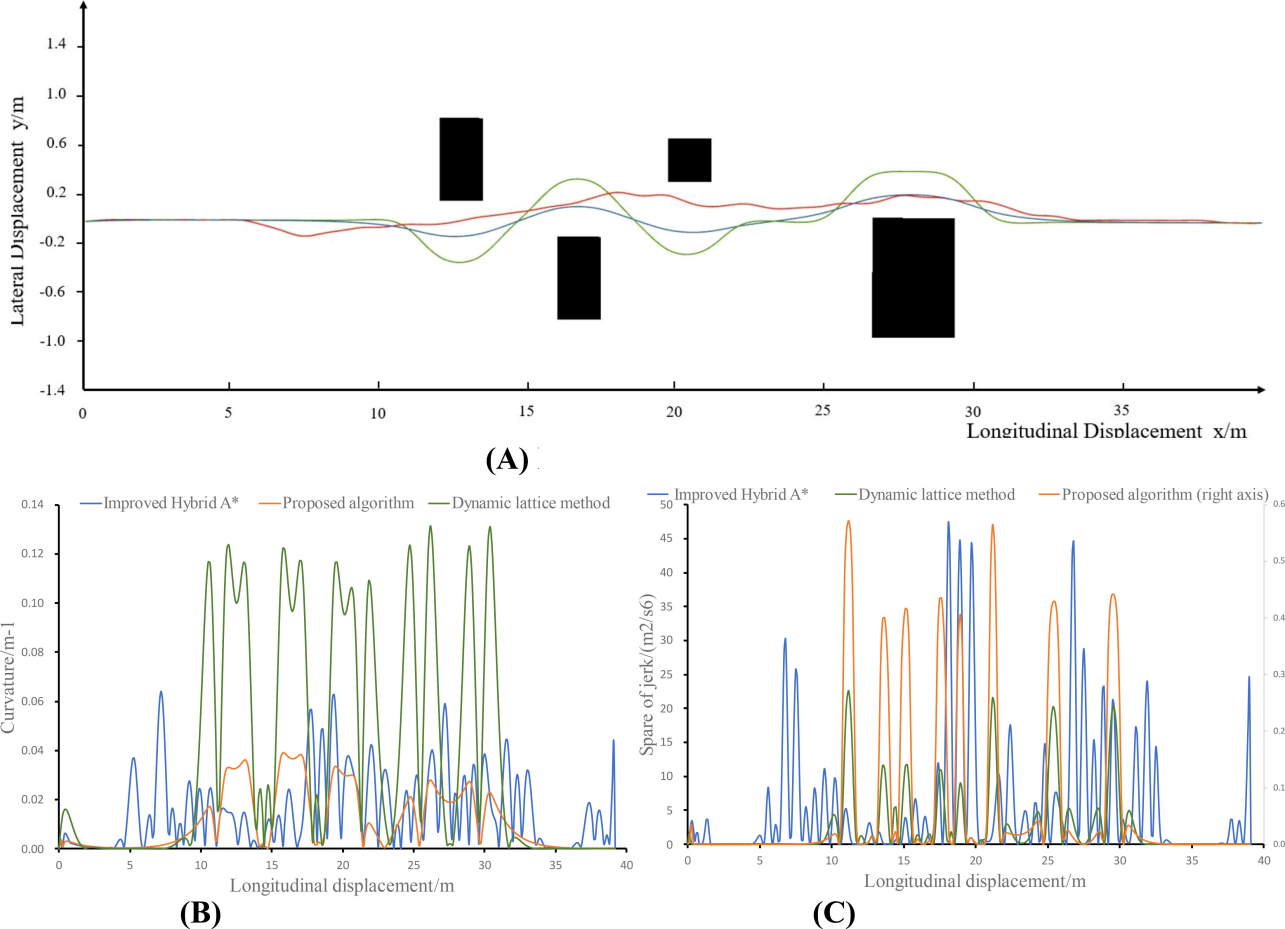
Simulation experiment results. **(A)** Path planning results. **(B)** Planning path curvature. **(C)** Planning path jerk.

The experimental results are shown in **Figure 7** and **Table 1**, where a is the result of the planned trajectory and b, c, and d are the curvature, jerk, and control error changes of the planned path, respectively.

**Table 1:** Comparison of the planning paths.

method		Path deviation (m)	Average curvature ( $m^{-1}$ )	Integral of jerk² $\left(m^{2}/s^{6}\right)$	Computation time (s)
Improved hybrid A* (Dolgov et al., 2010)	mean	0.102	0.02	2008.00	0.2592
	max	0.224	0.06	2920.00	
Dynamic lattice method (Fan et al., 2018)	mean	0.123	0.02	364.882	0.027
	max	0.437	0.15	524.50	
Proposed method	mean	0.075	0.01	103.473	0.0098
	max	0.176	0.07	107.50	

As shown in the table, all three methods generate kinematically feasible trajectories. However, the method proposed in this paper is an order of magnitude faster and tends to generate a path that deviates less from the original route but is smoother while maintaining a curvature that is at a minimum and does not exceed the maximum curvature limit and an order of magnitude lower acceleration change rate. In the proposed method, deviation, curvature, and jerk are all minimized as optimization terms, thereby facilitating the generation of superior-quality paths.

#### 3.2.2 Real machine test results and analysis

**Figure 8:**
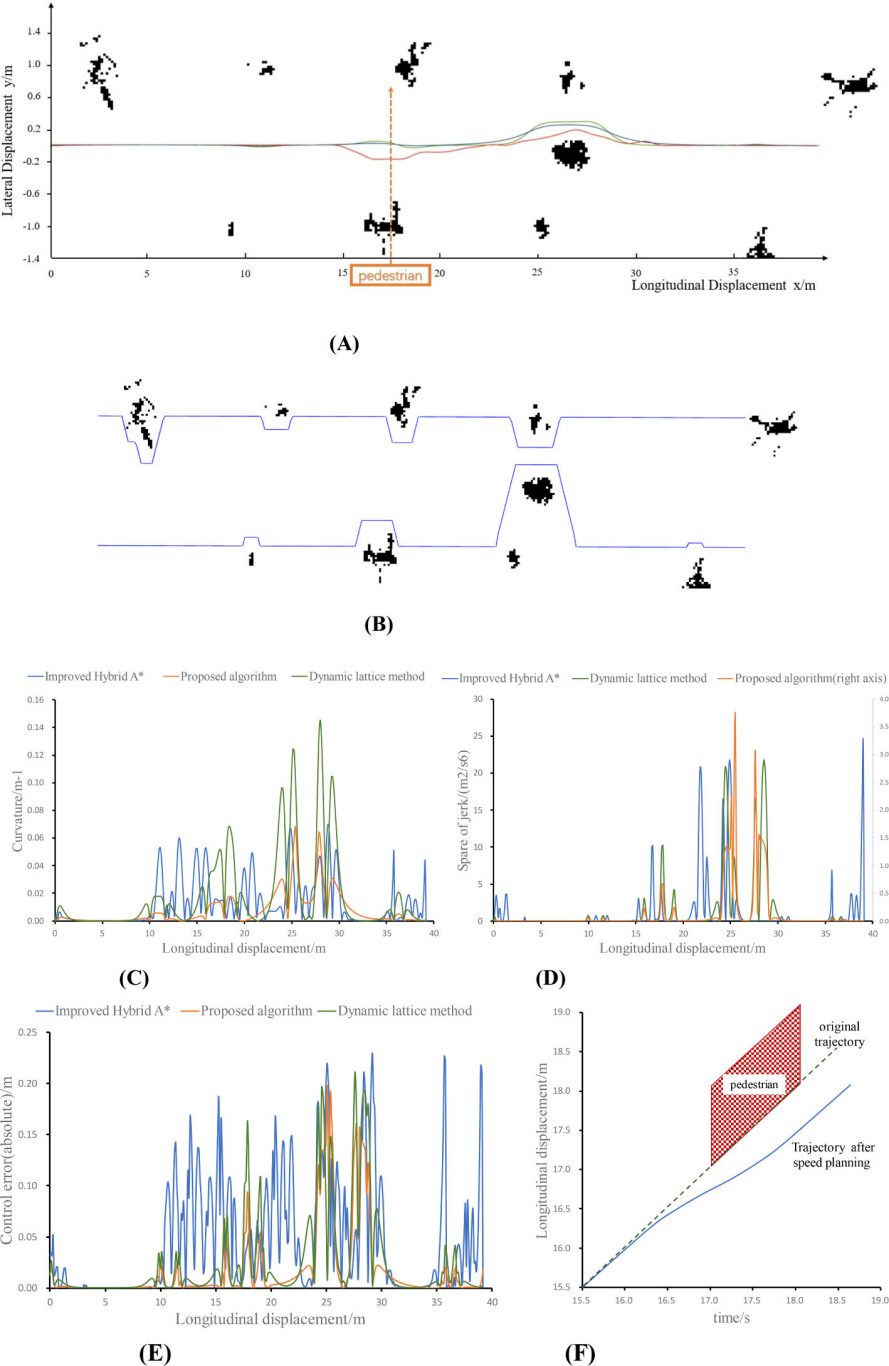
Experimental results for a real vehicle. **(A)** Path planning results, **(B)** Safe Corridor, **(C)** Planning path curvature, **(D)** Planning path jerk, **(E)** Absolute control error, **(F)** Result of speed planning.

The experimental results are shown in **Figure 8** and **Table 2**, where a is the result of the planned trajectory and b, c, and d are the curvature, jerk, and control error changes of the planned path, respectively.

**Table 2:** Comparison of the planning paths.

method			Path deviation (m)	Average curvature ( $m^{-1}$ )	Integral of jerk² $\left(m^{2}/s^{6}\right)$	Control error (m)	Computation time (s)
Improved hybrid A* (Dolgov et al., 2010)		mean	0.145	0.01	476.00	0.087	0.3881
		max	0.259	0.07	993.20	0.232	
Dynamic lattice method (Fan et al., 2018)		mean	0.131	0.01	164.06	0.0428	0.022
		max	0.476	0.07	287.16	0.0847	
Proposed method		mean	0.133	0.008	40.38	0.0373	0.0096
		max	0.246	0.05	102.00	0.0697	

In **Figure 8A**, the blue trajectory is planned by the algorithm proposed in this paper, the green trajectory is planned by the dynamic lattice method, and the red trajectory is planned by the improved hybrid A* method. Moreover, the pedestrian crosses the operation area at x = 17.

As shown in the data in the table, the algorithm proposed in this paper ensures the continuity of the mower path, speed and curvature changes, and at the same time, it automatically adjusts the longitudinal speed of the mower in the process of obstacle detouring. This guarantees the safety of the mower operation in terms of path planning and speed planning, which is favorable for the control of the mower. The algorithm proposed in this paper, which features a smaller curvature and lower degree of transverse motion, exhibited the lowest control error. In addition, due to the reduced search dimension, the algorithm proposed in this paper and the dynamic lattice method, which are lateral and longitudinal decoupling path planning algorithms based on the reference line, respectively, have a significant advantage in terms of computing time compared with the path planning algorithms under open space. The proposed safe corridor search method is characterized by a reduced number of arithmetic steps in comparison to dynamic programming. This results in the creation of a convex space for searching in quadratic programming. Once more, the rapidity of quadratic programming allows the method proposed in this paper to have a markedly lower computational time overhead in comparison to the other two methods. Consequently, the average computing time is reduced by 398.5 ms and 326.1 ms, respectively, which guarantees the timeliness and safety of the lawnmower to make a correct response when it encounters an obstacle.

## 4 Conclusion

The current open space and structured algorithms for local path planning in orchard mowing operations have shortcomings in terms of both computing efficiency and path quality. In this paper, we propose a local path planning method that utilizes safe corridors and quadratic programming. Moreover, depth-first search is implemented to determine detour directions, and safe corridors are constructed to provide a convex space for the optimization algorithm. Additionally, piecewise jerk and curvature limits are introduced in quadratic programming to ensure higher-order continuity and curvature feasibility of the path.

During real-vehicle tests, this method plans an obstacle avoidance path with an average curvature of 0.008 m-1 and generates an average lateral error of 3.73 cm during path tracking. The algorithm presented in this paper has an average consumption time of 9.6 ms, which is a significant improvement compared to the dynamic lattice method and the hybrid A* algorithm, reducing the average time consumed by 12.4 ms and 387.4 ms, respectively. The algorithmic efficiency is improved by 129% and 4035%, respectively. The algorithm proposed in this paper plans an obstacle avoidance path that meets the maximum curvature requirement of the mower chassis. This enables the mower to smoothly and stably avoid stationary obstacles. Therefore, a new path planning method for the automatic operation of a mower is presented in this paper. We are continuously refining the pipeline of the method and will conduct tests on complex and diverse orchard scenarios in subsequent studies to improve its robustness and broad applicability.

## Data Availability

The raw data supporting the conclusions of this article will be made available by the authors, without undue reservation.
